# Motor Oil Classification Based on Time-Resolved Fluorescence

**DOI:** 10.1371/journal.pone.0100555

**Published:** 2014-07-02

**Authors:** Taotao Mu, Siying Chen, Yinchao Zhang, Pan Guo, He Chen, Fandong Meng

**Affiliations:** School of Optoelectronics, Beijing Institute of Technology, Beijing, China; CNR, Italy

## Abstract

A time-resolved fluorescence (TRF) technique is presented for classifying motor oils. The system is constructed with a third harmonic Nd:YAG laser, a spectrometer, and an intensified charge coupled device (ICCD) camera. Steady-state and time-resolved fluorescence (TRF) measurements are reported for several motor oils. It is found that steady-state fluorescence is insufficient to distinguish the motor oil samples. Then contour diagrams of TRF intensities (CDTRFIs) are acquired to serve as unique fingerprints to identify motor oils by using the distinct TRF of motor oils. CDTRFIs are preferable to steady-state fluorescence spectra for classifying different motor oils, making CDTRFIs a particularly choice for the development of fluorescence-based methods for the discrimination and characterization of motor oils. The two-dimensional fluorescence contour diagrams contain more information, not only the changing shapes of the LIF spectra but also the relative intensity. The results indicate that motor oils can be differentiated based on the new proposed method, which provides reliable methods for analyzing and classifying motor oils.

## Introduction

Motor oils, as products of petroleum refinery, are irreplaceable in car industry. They are used to protect the engine from many physically and chemically related malfunctions like heating, corrosion and contamination [1], [2]. Due to the large variations in price of motor oils, cheap substituent products instead of more expensive petroleum products have been used by some profit-driven businesses to maximize the interest. It arisen the question about quality authentication [1].

There are many studies about the petroleum [3]–[6]. However the studies concentrating on motor oils are rare. Recently motor quality authentication is gaining increasing attention. As to Roman M. Balabin, motor oil classification is an important task for quality control and identification of oil adulteration [7]. Over the past several years many new technologies have surged in motor oils identification. Due to the usage for exploratory analysis and classification, chemometric techniques are often used in oils analysis [1]. However, sample preparation is time consuming in the way described above. In addition, it is destructive analysis methods.

Liquid chromatography has been widely used in many fields including petroleum industry. [8]–[10] Dielectric spectroscopy is used in classification of engine oil by Guan, L. [11]. As a non-destructive measurement technique, near infrared (NIR) spectroscopy has been widely used in petroleum industry [12]–[16]. Near infrared (NIR) spectroscopy is used to classify motor oils by base stock and viscosity in Ref. [17]. The spectral range is from 780 nm to 1400 nm. A commercial IR spectrometer combined with multivariate data analysis is employed. The probabilistic neural network (PNN) method and classifiers have shown good results. But NIR measurements are scarcely selective, so it should be used together with chemometric techniques. And the low sensitivity is another disadvantage of IR [18].

In some recent years, fluorescence has been widely used in food [19], [20], medicine [21]–[23], petroleum industry [24]–[26]. The application of time-resolved fluorescence (TRF) has a development in petroleum industry [27]–[30]. TRF has also been used to characterize of crude oil in Ref. [31], [32]. The variation in the spectral profile of the emitted fluorescence bands as a function of time is measured in the experiment. Nine oil samples are successfully distinguished by this approach.

Experiments demonstrate that the TRF shapes of motor oil are distinct from each other. In this paper, Contour diagrams of normalized TRF intensities (CDTRFIs) are constructed to serve as fingerprints for classifying motor oils. The oils can be distinguished by the new presented methods. Superior to ordinary fluorescence spectra, the two-dimensional fluorescence contour diagrams of motor oils contain more information, not only the changing shapes of the LIF spectra but also the relative intensity [33]. Due to the non-destructive and high sensitive characteristic, CDTRFIs make identification more accurate and reliable.

## Materials and Methods

Oil samples are obtained from local market and stored in a dark room during the period of analysis. Nine motor oil samples of five popular brands (Table 1) and different SAE grades are used for classification in this paper.

**Table 1:** Motor oils used in the study.

No.	API service	SAE grade	company
1	SM	5W-40	Shell
2	SF	15W-40	Shell
3	SN	5W-40	Mobil
4	SN	5W-30	Mobil
5	SN	0W-40	Mobil
6	SF	20W-40	Mobil
7	SN/CF	5W-40	Castrol
8	SM	5W-40	Prestone
9	SJ	10W-50	GreatWall

Steady-state and time-resolved fluorescence spectra are collected and analyzed in the study. CDTRFIs are based on measuring the variations in the shapes of the TRF spectra at specific gate delay time (GDT) within the laser-pulse time profiles [33]. GDT should be set to 0 ns and the gate-width of ICCD should be set to 75 ns when steady-state fluorescence is collected. Experiments demonstrated that fluorescence intensity is too weaker to detect when GDT is larger than 75 ns. Before CDTRFIs constructed, GDT is increased from 0 ns to 75 ns by intervals of 5 ns. Thus, 16 independent fluorescence spectra, used to construct CDTRFIs, are collected for each oil sample. Background and noises are removed from all the fluorescence spectra at emission wavelengths (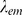) between 360 and 675 nm. In order to avoid the geometrical effects on fluorescence measurement, we normalize the maximum fluorescence intensities to 1. All the measurements are repeated three times to ensure the repeatability of this approach.

## Experimental setup

**Figure 1:**
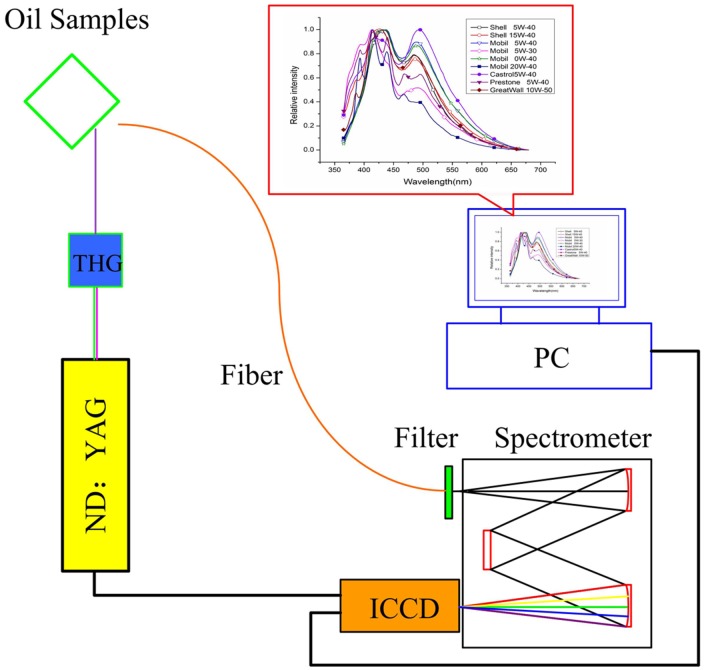
Schematic of the experimental set-up. PC: personal computer; Filter: 355 nm long-pass filter; ICCD: intensified charge-coupled device; THG: third harmonic generator output at 355 nm.

Figure 1 presents the configuration of experimental setup. a third-harmonic Nd:YAG laser (laser repetition rate 10 Hz, pulse width 3 ns, and pulse energy 40 mJ at 355 nm.) is employed. Motor oil samples are placed in a 10 mm fused-quartz cuvette. Front face illumination is used in the study to decrease inner effect, which is caused by high optical densities. A quartz optical fiber (NA = 0.22) are used to collect fluorescence directly. To block the strong elastic light from reaching the fluorescence detector, a 355 nm long-pass filter (Semrock BLP01-355R; cutoff wavelength of 361 nm) is inserted before the entrance slit (0.1 mm) of spectrometer. An intensified charge-coupled device (ICCD) camera (LI2CAM, Lambert Instruments) is placed behind the spectrometer to detect the dispersed fluorescence. The light is finally focused through the entrance slit and dispersed by a spectrometer (spectral range from 360 nm to 675 nm). On one hand, ICCD shows high sensitivity of a single photon level combined with fast gating of less than 3 ns. On the other hand, ICCD contain an embedded digital delay/pulse generator, which can synchronize laser and ICCD itself without any other equipment. A computer is employed to sample and digitize the acquired fluorescence and finally complete experiment data processing and results analysis.

## Results and Discussion

### 0.1 Steady-state Fluorescence Spectra of Motor Oil

**Figure 2:**
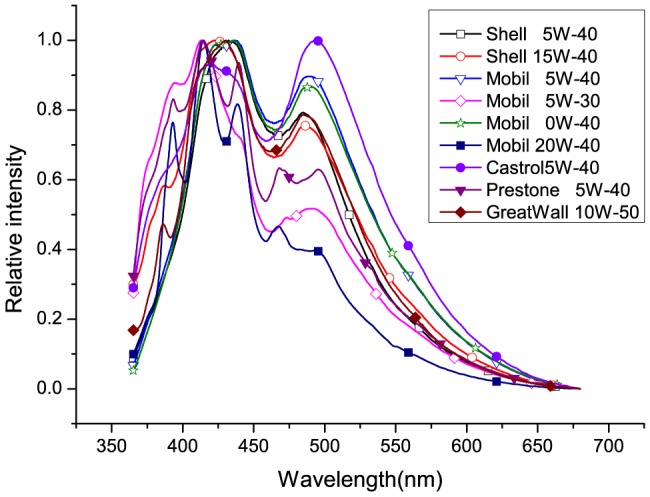
Normalized steady-state fluorescence spectra of motor oils, including, Shell 5W-40, Shell 15W-40, Mobil 5W-30, Mobil 5W-40, Mobil 0W-40, Mobil 20W-40, Castrol 5W-40, Prestone 5W-40, GreatWall 10W-50, under 355 nm laser pulse excitation.

The normalized steady-state LIF spectra of nine motor oils (including Shell 5W-40, Shell 15W-40, Mobil 5W-30, Mobil 5W-40, Mobil 0W-40, Mobil 20W-40, Castrol 5W-40, Prestone 5W-40, and GreatWall 10W-50) are shown in Figure 2. The excitation wavelength (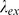) is 355 nm. The GW is set to 75 ns, approximately including all the fluorescence emitted by the samples. However, some of the normalized fluorescence spectra (Figure 2) are similar at one excitation wavelength only (355 nm). Four main characteristic peaks (centered at 380 nm, 410 nm, 435 nm, and 490 nm) could be found in Figure 2.

### 0.2 Influence of GDT on Fluorescence Spectra

**Figure 3:**
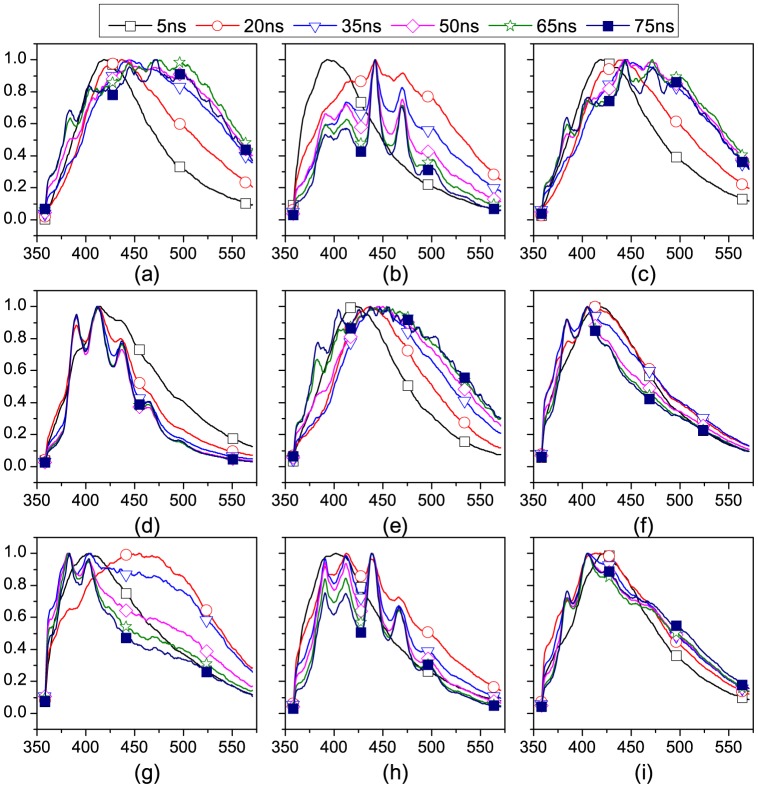
LIF spectra at specific time gates (including 5 ns, 20 ns, 35 ns, 50 ns, 65 ns, 75 ns). (a) Mobil 0W-40, (b) Mobil 5W-30, (c) Mobil 5W-40, (d) Mobil 20W-40, (e) Shell 5W-40, (f) Shell 15W-40, (g) Castrol 5W-40, (h) Prestone 5W-40, and (i) GreatWall 10W-50. The normalized fluorescence intensity (y-axis) and the wavelength (x-axis) are used as axes. The wavelength range is 360 nm to 575 nm. The integration time is set to 5 ns.

The normalized LIF spectra of different GDT (5 ns, 20 ns, 35 ns, 50 ns, 65 ns, and 75 ns) of oil samples are presented in Figure 3 (The data of oils can be found in Data S1). The GW is set to 5 ns. AS GDT is increased from 0 ns to 75 ns, the shapes of fluorescence of motor oils change a lot. Some new characteristics peaks appear while some old peaks disappear. And the intensity of each peak varies as the GDT increasing. As to E. HEGAZI, because not all the excited aromatic compounds have the same lifetime, the fluorescence spectrum of any oil will have different shapes when measured at different time windows [31]. Then time-character will reflect in the fluorescence when GW is narrower that fluorescence lifetime. The fluorescence spectrum should be correlated with GDTs. Equation 1, 2 can be derived [33]:
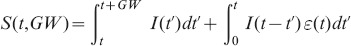
(1)

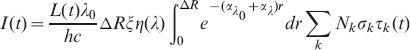
(2)


Where:


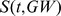
 is the fluorescence photon numbers collected by detector at GDT 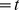 with gate width = GW, Including excited directly by the laser and other relaxation of energy transfer processes.


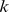
 refers to any compounds capable of fluorescing at 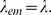



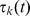
 is the time distribution function of fluorescence of compounds. 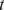 is detection time 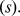



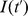
 is the fluorescence excited directly, which occurs within the 5 ns GW.


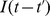
 is the fluorescence excited at earlier time, which will encounter a decay according to some exponential function 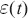



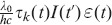
 is the reciprocal of laser photo energy 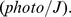



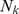
 is the concentration of each compound 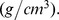



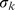
 is fluorescence cross section of different compounds 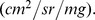



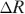
 is optical path in the sample 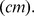



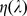
 is total receiver efficiency at 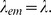



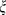
 is the geometric overlap factor.


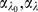
 are extinction coefficient at 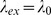 and 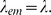


These variations in the spectra will be different for different oils, as we will see shortly, and can be conveniently taken advantage of to characterize these oils.

### 0.3 Contour Diagrams of TRF Intensities of Motor Oils

**Figure 4:**
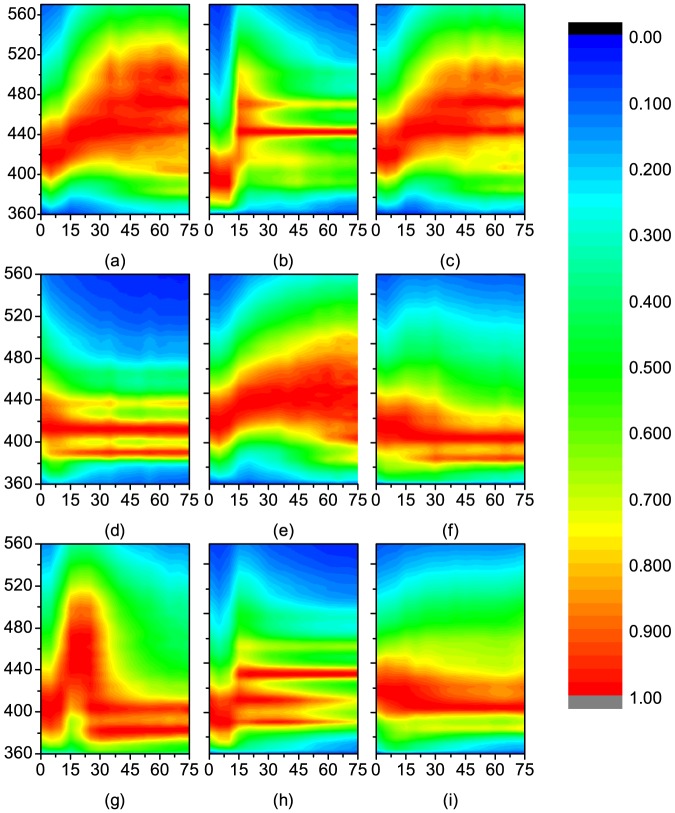
CDTRFIs of (a) Mobil 0W-40, (b) Mobil 5W-30, (c) Mobil 5W-40, (d) Mobil 20W-40, (e) Shell 5W-40, (f) Shell 15W-40, (g) Castrol 5W-40, (h) Prestone 5W-40, and (i) GreatWall 10W-50. The fluorescence wavelength (y-axis) and the detection time (x-axis) are used as axes. The wavelength range is 360 nm to 560 nm, and the time range is 75 ns with an excitation wavelength of 355 nm. The gate width of the ICCD is set to 5 ns.

CDTRFIs are constructed based on the time-character of LIF of motor oils (Figure 4, The data of oils can be found in Data S1). Wavelength and GDTs are used as axis of CDTRFI to show the variation of fluorescence spectra shapes over time. It should be pointed out that the zero point of the GDT (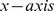) is set when the sample illuminated by laser. GDTs increase from 0 ns to 75 ns with a 5 ns sampling interval. The spectra (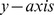) range from 360 nm to 675 nm at only one excitation wavelength (355 nm). Contour diagrams are constructed in this way when all the spectra are normalized. The most important parameter of CDTRFIs is the increment between the contour lines. The smaller the increment, the more contour features will be revealed and the higher the resolution of the fingerprints will become. However, the limit of the increment will depend on the uncertainty of the TRF spectra [31]. The increment is set to 0.02 in this study.

Then fluorescence should be measured at 16 different GDTs for each oil to construct intact CDTRFI. To eliminate the influence of energy fluctuation, this process should be repeated at least three times. It is found that CDTRFIs, showing not only the variation in the shape of TRF spectra over time (along 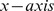) but also a series of normalized LIF spectra of different GDTs (along 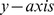), can serve as unique fingerprints for motor oils. The CDTRFIs of motor oils are quite different from each other, suggesting that the effective of TRF-based method. Outperform to the steady-state LIF, the distinction among the CDTRFIs of different oils is more significant. The Motor oil samples can be easily distinguished by this method, while it is hard to classify all the oil samples just by steady-state LIF fluorescence spectra. All the measurements are repeated three times to ensure the repeatability of this method.

In this paper, the contour diagrams are constructed at only one excitation wavelength 355 nm. The CDTRFIs of motor oils providing additional information of motor oils to enhance the recognition rate.

## Conclusions

A new method based on TRF is proposed for motor oils classification. The LIF spectra of nine kinds of motor oils are measured at 16 different GDTs in the study. Then CDTRFIs are constructed by using these spectra measured at different GDTs. Superior to steady-state LIF, this approach presents not only a series of normalized fluorescence spectra (along 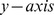) but also the variation in the spectra shapes (along 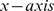) as the GDTs increasing. The proposed method is shown successful in classification of nine motor oils, and to be capable in distinguishing between more similar oils. The method may be further improved by reducing the GW of the ICCD and by using smaller increments in the counter. Considering the high sensitive and non-destructive ability of CDTRFIs, the presented method can be used as unique fingerprints of motor oils, providing researchers with a reliable means of characterizing and distinguishing motor oils.

## Supporting Information

Data S1 **The time-resolved fluorescence data of motor oils.** (Including Mobil 0W-40, Mobil 5W-30, (c) Mobil 5W-40, Mobil 20W-40, Shell 5W-40, Shell 15W-40, Castrol 5W-40, Prestone 5W-40, and GreatWall 10W-50). The wavelength range is 360 nm to 560 nm, and the time range is 75 ns with an excitation wavelength of 355 nm. The gate width of the ICCD is set to 5 ns.
(ZIP)
